# How Does Leader Humility Influence Team Creativity? The Roles of Team Behavioral Integration and Leader Performance

**DOI:** 10.3389/fpsyg.2022.818865

**Published:** 2022-05-06

**Authors:** Taiqiu Zhu, Yixuan Chen, Eric Adom Asante, Yue Zhu, Tingting Xu

**Affiliations:** ^1^School of Business Administration, Zhejiang Gongshang University, Hangzhou, China; ^2^Lee Shau Kee School of Business and Administration, Hong Kong Metropolitan University, Hong Kong, Hong Kong SAR, China

**Keywords:** leader humility, team behavioral integration, team creativity, leader performance, social learning theory

## Abstract

This study developed and tested a research model to examine the influence of leader humility on team creativity. Drawing on social learning theory, we tested team behavioral integration as a mediator in the relationship between leader humility and team creativity. Moreover, we tested the moderating effect of leader performance on this mediated relationship. We tested our hypotheses using a multiple-source research design. Data were collected from 275 employees in 67 work teams from a variety of industrial companies in Southeast China. The results confirmed that team behavioral integration mediated the relationship between leader humility and team creativity. Furthermore, the indirect effect of leader humility on team creativity *via* team behavioral integration was stronger when leader performance was higher (vs. lower). We discuss the implications of our findings for the theory and practice of leader humility.

## Introduction

For the most part, both the media and extant leadership research have portrayed leaders as heroes, faultless, and individuals who see themselves in an overly positive light (Chatterjee and Hambrick, 2011; Park et al., 2011). The narrative is that such leaders do not accept blame or appreciate followers’ efforts and contributions. The arrogance, sense of entitlement, and narcissistic tendencies of such leadership style have been attributed to why leaders make bad decisions (Dotlich and Cairo, 2003; Chatterjee and Hambrick, 2007). As a result, scholars have called for other forms of leadership whereby leaders acknowledge their limitations and recognize their followers’ strengths (Owens and Hekman, 2012). Indeed, as the work environment becomes more turbulent, complex, and uncertain (Crossan et al., 2008), it becomes difficult for a single dominant leader (e.g., a paternalistic leader, for whom authoritarianism is an important characteristic) to solve all problems from the top (Owens and Hekman, 2016). One type of bottom-up leadership style that has been proposed to help organizations adjust to the fast-changing work context is humble leadership. Humble leaders encourage the initiatives and input of their team members, and this has been found to improve team outcomes such as performance and effectiveness (e.g., Owens et al., 2013; Chiu et al., 2016; Owens and Hekman, 2016; Rego et al., 2017a).

Recently, scholars have found that humble leadership is also important in promoting team creativity (Hu et al., 2018; Wang et al., 2019; Chen et al., 2020), defined as “the production of novel and useful ideas concerning products, services, processes and procedures by a team of employees working together” (Shin and Zhou, 2007, p. 1710). Because humble leaders are open to followers’ ideas and feedback (Cheung et al., 2020), they have been found to encourage team creativity through team information sharing and team psychological safety (Hu et al., 2018). However, some scholars and practitioners are skeptical about the effectiveness of humble leadership, given that it is contrary to the normal sense of leadership, including the exertion of power and authority (Owens and Hekman, 2012). They argue that humble leaders may not be effective because humility can suggest incompetence or weakness to their followers.

To examine this issue, we draw on social learning theory (Bandura, 1977) to examine when and under what conditions humble leadership is effective in influencing team creativity. First, we propose team behavioral integration as an important mechanism through which leader humility affects team creativity. Team behavioral integration refers to the degree of mutual and collective interaction between group members (Hambrick, 1997). Team behavioral integration combines a social aspect and two task aspects of the group process (Simsek et al., 2005). The social aspect is the team’s collaborative behavior, and the task aspects are information exchange and joint decision making (Simsek et al., 2005). Unlike other team process constructs such as social integration and information sharing, team behavioral integration connotes the relationships between team members and offers a well-developed and useful construct for exploring processes and dynamics within teams (Carmeli and Waldman, 2009). We argue that because humble leadership encourages followers to share their ideas and opinions (Li et al., 2019), such leaders engender behavioral integration in their team members that in turn leads to high team creativity.

Second, some scholars have suggested that humble leadership can be viewed as incompetence, thus weakening the effectiveness of humble leaders (Exline and Geyer, 2004; Judge et al., 2009). As the behavior of humble leaders is likely to be attributed to their shortcomings, employees may “associate humility with failure experiences that are depressing or threatening to recall, and even associate humility with interpersonal risks” (Exline and Geyer, 2004, p. 98). This suggests that the factors that can cause followers to doubt their leaders’ competence may act as a boundary condition for the effectiveness of humble leadership. However, few studies have considered such leader-related variables. Therefore, we propose leader job performance as a potential moderator, because high job performance can indicate a leader’s underlying competence (Lord and Dinh, 2014). Furthermore, as an explicit indicator, job performance is relatively easy for employees to observe. In this way, we seek to establish how leaders can be humble and convey positive messages simultaneously.

**Figure 1:**
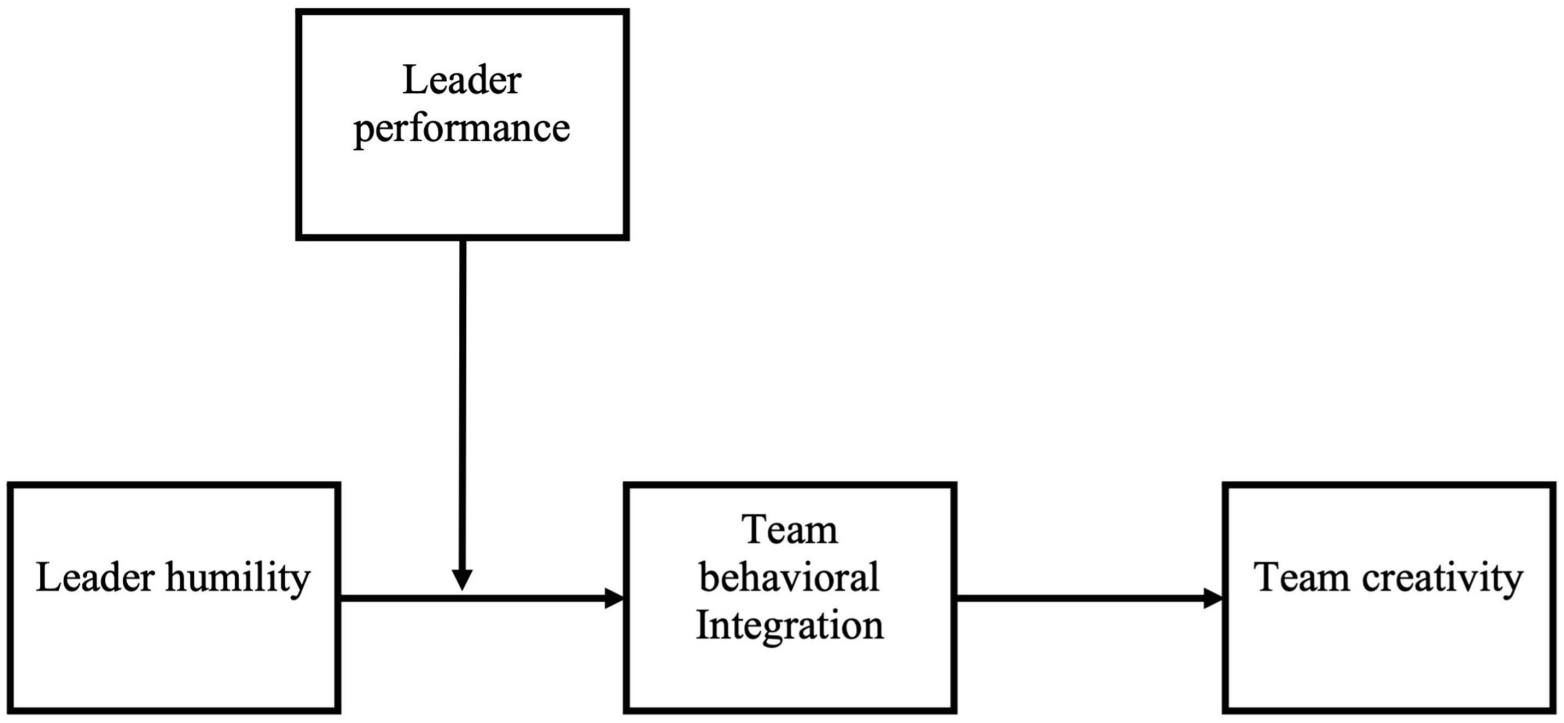
The hypothesized model.

This study contributes to the literature on humble leadership in two important ways. First, some studies agree that humble leadership is important for team creativity (e.g., Chen et al., 2020). However, others are equivocal about the relationship between the effectiveness of humble leadership and team outcomes (e.g., Owens and Hekman, 2012). We contribute to this stream of research by examining how and when humble leadership leads to team creativity. Second, given that humble leadership contradicts the normal meaning of leadership that connotes power and status (Owens and Hekman, 2012), some followers may perceive humble leaders as weak or incompetent. We examine the role of leader performance as a competency condition that enhances the effect of leader humility. We show that when humble leaders achieve high performance, their followers see them as competent, which enhances their influence on team behavior. Our hypothesized model is presented in Figure 1.

## Literature Review and Hypothesis Development

Leader humility was once considered as a specific trait possessed by some leaders (Tangney, 2000), but it is now more often regarded as a behavior that is easy to observe and imitate. Owens et al. (2013) defined leader humility as “an interpersonal characteristic that emerges in social contexts that connotes (a) a manifested willingness to view oneself accurately, (b) a displayed appreciation of others’ strengths and contributions, and (c) teachability” (p. 1518). When leaders interact with others, humility can be manifested through their behavior. Specifically, humble leaders are willing to accept their mistakes and shortcomings in addition to their abilities. Humble leaders also admire the strengths of others and appreciate their work commitment (Owens and Hekman, 2012). They are open to feedback and different ideas and are willing to change or strive to improve themselves (Morris et al., 2005). Although humble leadership may seem similar to moral leadership (e.g., ethical leadership, servant leadership, and benevolent leadership), it differs in certain respects. For instance, ethical leaders use punishment to reinforce followers’ behavior (Treviño et al., 2003; Brown et al., 2005), whereas humble leaders show great respect. Moreover, unlike servant leaders, humble leaders use modeling to demonstrate to followers how to grow and learn rather than merely serve others, which may imply uncertain outcomes (e.g., psychological freedom for both leader and follower and fluidity in organizing; Owens and Hekman, 2012). Benevolent leaders show genuine caring and protection and focus on the welfare of their employees (Pellegrini and Scandura, 2008) but neglect the importance of accurate self-awareness, which is a key element of humble leadership. In sum, humble leaders view themselves objectively and accurately, regard others appreciatively, and remain open to new information and ideas (Owens and Hekman, 2012). Given the positive and interpersonal nature of humble leadership, research has shown empirically that leader humility has a great impact on team processes (e.g., information sharing, Hu et al., 2018) and team performance (e.g., Rego et al., 2017b).

This study extends this line of research by arguing that humble leaders can influence team behavioral integration and in turn increase team creativity. Team behavioral integration, defined as the extent to which team members mutually and collectively interact with each other, has been shown to have three components: information exchange, collaboration, and joint decision making (Hambrick, 1997). In addition to information exchange, high behavioral integration means that members share resources, assistance, and cooperation (Gu et al., 2016). Further, team members typically have a shared understanding of the problems they face and discuss and make joint decisions (Halevi et al., 2015). Although team behavioral integration was first proposed in the context of top management teams, recent studies have tested and demonstrated the validity of this construct in common workgroups (e.g., Carmeli and Waldman, 2009; Sousa and Van Dierendonck, 2016; Tekleab et al., 2016).

Drawing on social learning theory (Bandura, 1977), we argue that leader humility may promote team behavioral integration. Social learning theory emphasizes that individuals can form integrated behavioral patterns by observing the behavior of others and its consequences (Bandura, 1977). Moreover, from the social learning perspective (Bandura, 1977), modeling is indispensable in producing certain complex behaviors and can save time in acquiring such behaviors. Social cues from others guide individuals to follow desired behavioral norms and meet expected performance standards (Ou et al., 2014). As leaders have high status and authority, their behavior serves as the most salient information source and role model to guide interpersonal behavior (Huang, 2019; Qian et al., 2020). Therefore, based on social learning theory, we argue that when leaders consistently demonstrate humble behavior, employees try to interact with similar humility and exhibit a high level of team behavioral integration.

### Leader Humility, Team Behavioral Integration, and Team Creativity

As suggested by social learning theory (Bandura, 1977), humble leader behavior consciously or unconsciously models to team members that they should view themselves objectively, view others appreciatively, and show openness to new information and ideas. By observing and learning from humble leaders, team members can gradually develop highly integrated team behavior.

Specifically, we argue that humble leaders are likely to foster behavioral integration among team members. Humble leaders admire others’ opinions and appreciate their contributions and work devotion (Owens and Hekman, 2012), and this may lead to team behavioral integration in three ways. First, through daily interactions with members, humble leaders help members recognize their own unique strengths and expertise (Bandura, 1977). This builds team members’ self-efficacy in sharing ideas and expertise within the team and their belief that their contributions will be appreciated and accepted by their leader. Furthermore, humble leaders tend to admit their own limitations and be open to new ideas (Owens and Hekman, 2012). Members exposed to this behavior gradually learn to pay attention to their own weaknesses and shortcomings and become receptive to others’ ideas. Therefore, to improve themselves, members may proactively seek and exchange information with other members, thus exhibiting a high level of behavioral integration within the team.

Second, humble leaders encourage and empower members by admitting their own limitations and mistakes, recognizing members’ contributions and strengths, and showing teachability rather than simply giving directions. Through this behavior, humble leaders create a “cooperative, others-oriented interactive logic” within teams (Owens and Hekman, 2016, p. 1091) and model the prioritization of collective goals over personal status-seeking. Consequently, members collaborate to accomplish collective goals and are thus more willing to provide and seek mutual assistance (Carmeli et al., 2011), thereby enhancing behavioral integration within the team.

Third, humble leaders may create a platform for team members to make joint decisions. Given their clear self-awareness (Owens et al., 2013), humble leaders may realize that their managerial decisions are not always correct. Thus, to reduce potential mistakes and learn from members, they are likely to seek advice from team members to make better decisions for their team (Ou et al., 2014). Moreover, humble leaders admit their reliance on members, discuss team problems, and listen to members’ perspectives (Ou et al., 2014). These behaviors help members form shared perceptions of team goals and problems, further encouraging them to participate in team decisions. Based on the foregoing arguments, we propose the following hypothesis:

*Hypothesis 1*: Leader humility is positively related to team behavioral integration.

Team behavioral integration, in which the whole team shares information, resources, and decisions (Hambrick, 1997), is conducive to team creativity for several reasons. First, behaviorally integrated teams are likely to have a broad scope of information and perspectives that provide a cognitive base for members to generate novel and useful ideas (Amabile, 1996; Leung and Wang, 2015; Hu et al., 2018). Additionally, team behavioral integration fosters member commitment to collective goals and mutual collaboration to resolve team problems (Carmeli et al., 2011). When team members have high-level interactions, they are likely to effectively manage different and even conflicting creative viewpoints (Jaskyte, 2008). Members tend to transmit their diverse information, knowledge, and views into more novel and synthetic ideas that are useful for team innovation (Jahanshahi and Brem, 2017). In contrast, with low behavioral integration, team members are less able to integrate diverse ideas that contribute to team creativity. Furthermore, teams with high behavioral integration encourage collaborative behavior, which has been found to engender creativity in organizations (Rosa et al., 2008). A collaborative culture can inspire a team to be creative (Barczak et al., 2010).

Moreover, team behavioral integration can provide a supportive and trustful social environment (Lubatkin et al., 2006) for team creativity. Creativity usually entails risk because novel ideas may challenge the status quo within a team and are less likely to be accepted by leaders and other members (Farh et al., 2010). In this regard, members of behaviorally integrated teams are more likely to seek out and be receptive to the novel views and opinions of others (Hambrick, 1997). Furthermore, believing that their ideas will be respected by others, members are more likely to share their creative and unique ideas with the team, thus facilitating team creativity. At the empirical level, research has demonstrated that team behavioral integration is positively related to team creativity. For instance, among a sample of Iranian enterprises, Jahanshahi and Brem (2017) found that highly (vs. poorly) behaviorally integrated teams were more likely to inspire their members to be creative and propose more creative ideas. Based on these arguments, we propose that humble leaders facilitate behavioral integration within teams, which in turn promotes team creativity.

*Hypothesis 2*: Team behavioral integration mediates the relationship between humble leader behavior and team creativity.

### The Moderating Effect of Leader Performance

As mentioned, humble leaders send salient behavioral cues to shape members’ perceptions of the norms and expected behavior in the work context, and members tend to behave accordingly, i.e., by showing team behavioral integration in our study. Although the positive effects of leader humility have been widely demonstrated, researchers have also noted that “leader humility can sometimes be construed as weakness or low self-esteem” (Wang et al., 2018, p. 1023), which can cause members to react less positively to leader humility (Tangney, 2000). Following this notion, we argue that the positive effect of leader humility on team behavioral integration is contingent on whether members regard leader humility as an expression of weakness. Specifically, we introduce leader performance as a variable that can influence how members interpret humble leadership behavior. As high-performing leaders are generally perceived as highly competent (Yun et al., 2007), their team members are less likely to attribute their humility to weakness or low self-esteem. Instead, members may interpret it as a benevolent quality because leaders have no other reason for such behavior. This helps foster a trusting relationship between leaders and members, reinforcing the tendency of members to follow their leader’s social cues. In addition, the congruence of leader competence (connoted by leader performance) and virtue (connoted by humility) can help leaders gain high levels of respect from team members (Cheng et al., 2013; Bai et al., 2020). As social learning theory (Bandura, 1977) suggests, team members tend to learn from leaders who are highly respected. Consequently, high-performing humble leaders are likely to have a stronger influence on team behavioral integration.

In contrast, when leaders show low performance, members may interpret their humility as a sign of weakness or even as an apology for their failure to perform (Owens and Hekman, 2012). In this case, members may doubt their leader’s authority (Wang et al., 2018) and thus not conform or follow their leader’s instructions, such as by not participating in joint decision making. In addition, low leader performance may signal to members that humble behavior, such as seeking advice and assistance from others, is not beneficial to performance. Members then do not see humility as a useful strategy for improving their own performance, which reduces their motivation to imitate their leader’s behavior, thus weakening the effect of leader humility on team behavioral integration.

Based on these arguments, we propose the following moderation hypothesis:

*Hypothesis 3*: Leader performance moderates the relationship between leader humility and team behavioral integration, such that this positive relationship is stronger when leader performance is higher (vs. lower).

Combining the preceding arguments, we further propose a moderated mediation model. That is, when leader performance is high, humble leaders have a stronger modeling effect on team behavioral integration and, indirectly, on team creativity. However, when humble leaders show low performance, their influence on team behavioral integration and team creativity is weak.

*Hypothesis 4*: Leader performance moderates the indirect relationship between leader humility and team creativity through team behavioral integration, such that this indirect relationship is stronger when leader performance is higher (vs. lower).

## Materials and Methods

### Participants and Procedure

We used a simple sampling approach to collect data from several industrial companies in Southeast China. The companies’ HR managers were asked to serve as research assistants for the data collection. They were asked to contact a selection of teams from their companies to fill out the questionnaires. Eighty-five leaders with 350 employees volunteered to participate in the survey. Before delivering the questionnaires, all of the research assistants were trained in data collection procedures (e.g., standardized instructions). Leaders and employees received the survey questions on site with a cover letter guaranteeing confidentiality by the research assistants. The leaders responded to the scale on team creativity and provided their demographic data, while the employees evaluated their leader’s humility, the team’s behavioral integration, and their leader’s performance and provided their demographic details. They were required to return their completed questionnaires directly to the research assistants in a sealed envelope to ensure confidentiality.

After excluding incomplete or problematic questionnaires (e.g., those with too much missing data), our final sample consisted of 67 leaders and 275 employees (a response rate of 78.82% for leaders and 78.57% for employees). On average, each leader rated 4.10 employees (ranging from 3 to 7). Most employees (82.5%) were aged 20 to 40 years, and 60.4% were male. In terms of organizational tenure, 25.8% had less than 5 years, 30.2% had 6 to 10 years, 18.9% had 11 to 15 years, 17.5% had 16 to 20 years, and 7.64% had more than 20 years. In terms of education, 25.8% had a middle school diploma, 30.2% had a high school diploma, 18.9% had a technical college or vocational degree, and 25.1% had an undergraduate or graduate degree. Among the 67 leaders, most (68.6%) were aged 30 to 50 years, and 62.7% were male. In terms of organizational tenure, 19.4% had less than 5 years, 29.9% had 6 to 10 years, 22.4% had 11 to 15 years, 20.9% had 16 to 20 years, and 7.46% had more than 20 years. In terms of education, 28.4% of the leaders had a high school diploma, 34.3% had a technical college or vocational degree, and 37.3% had an undergraduate or graduate degree.

### Measures

We followed Brislin’s (1980) translation-back translation procedure to translate all English scales into Chinese. Unless otherwise specified, all measures were rated using a 5-point Likert-type scale ranging from 1 (*strongly disagree*) to 5 (*strongly agree*).

*Leader humility*. Leader humility was rated by the employees using the nine other-report items developed by Owens et al. (2013; *α* = 0.75). An example item is “My leader actively seeks feedback, even if it is critical.” In support of aggregation, the mean *r*_wg*(j)*_ across teams was 0.99, the interclass correlation (ICC[1]) estimate was 0.26, and the ICC(2) estimate was 0.59. Further, the one-way analysis of variance (ANOVA) showed significant differences in the team-level means of leader humility (*F* = 2.45, *p* < 0.01).

*Team behavioral integration*. Team behavioral integration was measured on the nine items developed by Simsek et al. (2005; *α* = 0.79). Following Carmeli and Waldman (2009), we substituted the word “work team” for “top management team” because our research focused on normal workgroups. The mean *r*_wg*(j)*_ across teams was 0.99, the ICC(1) estimate was 0.38, and the ICC(2) estimate was 0.72; the ANOVA results showed significant differences in the team-level means of team behavioral integration (*F* = 3.54, *p* < 0.01).

*Leader performance*. Leader performance was measured using the six items (*α* = 0.93) generated by Law et al. (2000). An example item is “My leader’s work quality is high with very few mistakes.” The median *r_wg(j)_* across teams was 0.97, the ICC(1) estimate was 0.53, and the ICC(2) estimate was 0.82. The ANOVA results showed significant differences in the team-level means of leader performance (*F* = 5.60, *p* < 0.01).

*Team creativity*. We measured team creativity using the four items developed and validated by Shin and Zhou (2007) (*α* = 0.74). An example item is “How well does your team produce new ideas?”

*Control variables*. We controlled for team size and average team tenure because these can potentially affect team processes and creativity (Dong et al., 2017; Mo et al., 2017; Zhang et al., 2019). We also controlled for leader gender and organizational tenure because research has suggested that these variables may influence employees’ expectations of leader humility (Eagly and Johnson, 1990; Ou et al., 2014).^1^

## Results

### Preliminary Analysis

We performed a multilevel confirmatory factor analysis on the key variables (i.e., leader humility, team behavioral integration, leader performance, and team creativity) to demonstrate construct validity. The hypothesized model was tested by loading the items on their respective latent variables at the within (leader humility, team behavioral integration, and leader performance) and between (leader humility, team behavioral integration, leader performance, and team creativity) levels. To achieve an optimal ratio of sample size to the number of estimated parameters, we reduced the number of indicators to the more parsimonious three parcels per latent factor by randomly averaging the items (Chin, 1998; Sass and Smith, 2006). The results showed good fit indices for the hypothesized model: *χ*^2^ (72) = 88.78, CFI = 0.99, TLI = 0.99, RMSEA = 0.03. The hypothesized model was further compared with a three-factor model with leader humility and leader performance loaded into a single factor at both levels. The results showed a significantly worse fit for the three-factor model (*χ*^2^ [77] = 659.74, CFI = 0.62, TLI = 0.50, RMSEA = 0.17; *∆χ*^2^ = 570.96, *p* < 0.01).

### Hypothesis Testing

Table 1 presents the means, standard deviations, and correlations for the variables. The correlation between leader humility and team behavioral integration was significant (*r* = 0.40, *p* < 0.01), providing preliminary support for Hypothesis 1. In addition, team behavioral integration was positively correlated with team creativity (*r* = 0.44, *p* < 0.01).

**Table 1:** Descriptive statistics and correlations among study variables.

Variable name	Mean	SD	1	2	3	4	5	6	7	8
1. Team size	4.10	1.05	–							
2. Average team tenure^a^	2.52	1.02	−0.03	–						
3. Leader gender	0.37	0.49	0.01	−0.36^**^	–					
4. Leader tenure^a^	2.67	1.22	0.16	0.35^**^	−0.58^**^	–				
5. Leader humility	3.82	0.23	0.23	−0.06	0.12	−0.10	(0.75)			
6. Team behavioral integration	4.20	0.29	0.10	−0.24^*^	0.10	−0.17	0.40^**^	(0.80)		
7. Leader performance	3.91	0.65	0.03	−0.04	0.01	0.02	−0.06	0.07	(0.93)	
8. Team creativity	4.67	0.37	0.01	−0.38^**^	0.05	−0.10	0.35^**^	0.44^**^	0.12	(0.74)

*N = 67 teams. Reliability values are in the parentheses along the diagonal*.

a *1 = less than 5 years, 2 = 5–10 years, 3 = 11–15 years, 4 = 16–20 years, and 5 = more than 20 years*.

* *p < 0.05*;

** *p < 0.01*.

The unstandardized path modeling results are presented in Table 2. In support of Hypothesis 1, leader humility was positively related to team behavioral integration (*B* = 0.48, *p* < 0.01). Further, team behavioral integration was positively related to team creativity (*B* = 0.36, *p* < 0.05). Hypothesis 2 predicted a mediating effect of team behavioral integration on the relationship between leader humility and team creativity. To examine Hypothesis 2, we used the PROCESS macro (Preacher et al., 2007) to derive the 95% CI of the indirect effect. The indirect effect of leader humility on team creativity *via* team behavioral integration was 0.17, with a 95% CI [0.018, 0.42]. Taken together, these findings supported Hypothesis 2.

**Table 2:** Unstandardized path modeling results.

Variables	Mediation model						Moderated mediation model				
	Team behavioral integration			Team creativity			Team behavioral integration			Team creativity	
	*Estimate*	*SE*		*Estimate*	*SE*		*Estimate*	*SE*		*Estimate*	*SE*
Intercept	2.64^**^	0.59		2.17^**^	0.78		4.44^**^	0.23		3.68^**^	0.71
Control variables											
Team size	0.01	0.03		−0.03	0.04		0.01	0.03		−0.03	0.04
Average team tenure	−0.06	0.04		−0.13^**^	0.04		−0.06	0.03		−0.13^**^	0.04
Leader gender	−0.05	0.09		−0.08	0.10		−0.05	0.08		−0.08	0.10
Leader tenure	−0.03	0.03		0.02	0.04		−0.02	0.03		0.02	0.04
Independent variables											
Leader humility	0.48^**^	0.15		0.40^*^	0.19		0.56^**^	0.15		0.40^*^	0.19
Leader performance							0.04	0.05			
Leader humility × Leader performance							0.39^*^	0.19			
Mediator											
Team behavioral integration				0.36^*^	0.15					0.36^*^	0.15
*R^2^*	0.22			0.34			0.28			0.34	

*N = 67 teams*.

* *p < 0.05*;

** *p < 0.01*.

**Figure 2:**
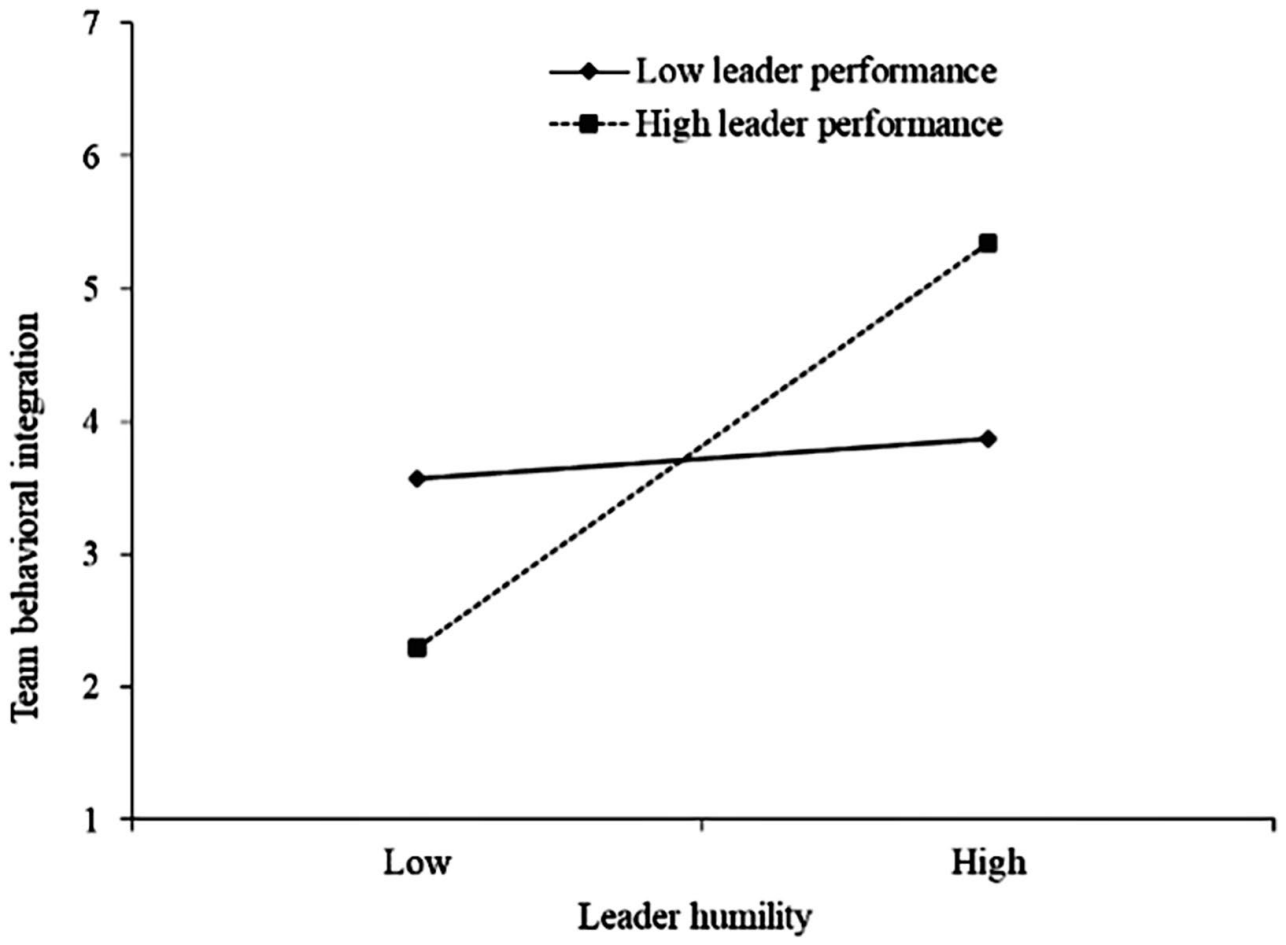
The interaction effect between leader humility and leader performance on team behavioral integration.

Hypothesis 3 predicts a moderating effect of leader performance on the relationship between leader humility and team behavioral integration, such that this positive relationship is stronger when leader performance is high. The interaction term between leader humility and leader performance was significantly related to team behavioral integration (*B* = 0.39, *p* < 0.05). The interaction pattern is plotted in Figure 2. When leader performance was high (+1 SD), the relationship between leader humility and team behavioral integration was stronger (simple slope = 0.81, *p* < 0.01) than when leader performance was low (−1 SD; simple slope = 0.31, n.s.). Thus, the findings supported Hypothesis 3.

To test the moderated mediation hypothesis (i.e., Hypothesis 4), we estimated the indirect relationship between leader humility and team creativity *via* team behavioral integration at higher and lower levels of leader performance. The results showed that when leader performance was high, the indirect effect of leader humility on team creativity *via* team behavioral integration was positive and stronger (indirect effect = 0.29, 95% CI = [0.05, 0.53]) than when leader performance was low (indirect effect = 0.11, 95% CI = [−0.001, 0.53]). Therefore, Hypothesis 4 was supported.

## Discussion

This study’s findings reveal that leader humility contributes to team creativity. The results also show that team behavioral integration mediates this relationship. This finding contributes to the understanding of how leader behavior affects team creativity through the behavioral integration of work teams. When leaders express humility, team members are likely to observe and learn humble behavior, which is reflected in their social and task processes and in turn promotes team creativity.

Moreover, drawing on social learning theory and the leader humility literature, we examined and demonstrated leader performance as an important environmental cue that moderates the effectiveness of leader humility. That is, when humble leaders had high performance, leader humility was positively related to team behavioral integration, whereas this relationship was nonsignificant when leader performance was low. Research has shown that followers mostly view and respond positively to humble leadership cues. However, recent studies have revealed some potential drawbacks of leader humility (e.g., Exline and Geyer, 2004; Qin et al., 2020). These studies argue that despite the widely reported positive effects of leader humility, it can also send behavioral cues that followers may misinterpret (Wang et al., 2018). The normative view of leaders is that they are egoistic and have a high sense of entitlement (Owens and Hekman, 2012). As a result, leaders who behave differently may be wrongly thought to be ineffective or weak, especially when contextual information (i.e., low leader performance in our study) guides followers to make such an interpretation. This faulty interpretation can undermine the potentially positive effect of leader humility on followers’ outcomes.

Finally, this study contributes to the literature on team behavioral integration by extending the discussion on the causes of team behavioral integration. Research has mostly relied on upper echelon theory to examine the antecedents of behavioral integration in top management teams (TMTs). It has been suggested that TMT members are likely to demonstrate team behavioral integration when the CEO has a collectivistic orientation (Simsek et al., 2005) and members identify with the team (Carmeli and Shteigman, 2010). In this study, we draw on social learning theory (Bandura, 1977) and reveal that followers can learn from humble leaders by observing and mimicking their behavior, leading to team behavioral integration, especially when the humble leader achieves high performance. Thus, we extend the team behavioral integration literature from the TMT level by identifying an important but neglected antecedent of behavioral integration in the workplace at the workgroup level.

### Practical Implications

We highlight the practical implications of leadership that expresses humility to enhance team processes and creativity. As humble behavior can be learned and expressed by leaders (Owens et al., 2013), companies should provide leadership training programs to help managers become humble leaders, such as by identifying their own strengths and shortcomings, recognizing employees’ contributions and advantages, and eliciting suggestions and ideas from employees. Moreover, we note that leader performance is a salient environmental cue for team members. Our study confirms that when humble leaders have high performance, team members are more likely to achieve high behavioral integration. Thus, a key component of effective humble leadership is the demonstration of high performance by leaders, which conveys a positive message to team members about the humble leader’s effectiveness.

### Limitations and Future Research

This study has several potential limitations that warrant consideration. First, team behavioral integration only considers the task and social aspects of integration to explain the team process. However, people also value emotional bonds, which could spur team members to contribute more to team creativity. A growing body of literature emphasizes trust as a key component when interpreting interpersonal and group behavior and managerial effectiveness (Hosmer, 1995). Thus, we suggest that future studies examine trust as a potential mediating mechanism in the effect of leader humility on team creativity.

Second, our results show that leader humility influences team behavioral integration, which in turn influences team creativity. However, we cannot draw a causal conclusion as our study is correlational rather than manipulative. Therefore, we call on future studies to use experimental approaches to test our model.

Finally, our study also has some limitations in collecting data. We only collected cross-sectional data by questionnaire from two cities in China to develop and test this model. To enhance the findings of our study and make it more generalizable, we recommend that future studies use a longitudinal design and a larger sample size from different districts or countries.

## Data Availability Statement

The raw data supporting the conclusions of this article will be made available by the authors, without undue reservation.

## Author Contributions

TZ was responsible for model development, methodology, data collection, and writing. YC and EA were responsible for writing and editing. YZ was responsible for data analyses and writing. TX was responsible for data collection. All authors contributed to the article and approved the submitted version.

## Conflict of Interest

The authors declare that the research was conducted in the absence of any commercial or financial relationships that could be construed as a potential conflict of interest.

## Publisher’s Note

All claims expressed in this article are solely those of the authors and do not necessarily represent those of their affiliated organizations, or those of the publisher, the editors and the reviewers. Any product that may be evaluated in this article, or claim that may be made by its manufacturer, is not guaranteed or endorsed by the publisher.

## Footnotes

^1^The significance and pattern of the results remained the same after excluding all of the control variables.
